# Perceived Social Support Moderates the Response to Stress and Alcohol Cues During Early Abstinence From Alcohol

**DOI:** 10.1016/j.bpsgos.2026.100762

**Published:** 2026-05-28

**Authors:** Samantha J. Goldstein, Rajita Sinha, Helen C. Fox

**Affiliations:** a Department of Psychiatry and Behavioral Health, Nassau University Medical Center, New York, New York; b Department of Psychiatry and Behavioral Health, Stony Brook University, Stony Brook, New York; c Department of Psychiatry, Yale Stress Center, Yale School of Medicine, New Haven, Connecticut; d Department of Psychiatry, Indiana School of Medicine, Indiana University, Indianapolis, Indiana

**Keywords:** Alcohol use disorder, Craving, Cue reactivity, HPA axis, Perceived social support, Stress reactivity

## Abstract

**Background:**

Alcohol addiction is associated with maladaptive stress responses that heighten craving and relapse risk during early abstinence. While perceived social support (PSS) is theorized to act as a stress buffer, its influence on acute stress responses during early abstinence remains poorly understood.

**Methods:**

Fifty-eight treatment-seeking individuals with alcohol dependence (45 males [77.6%] and 13 females [22.4%]) completed the Interpersonal Support Evaluation List (ISEL) upon entry into an inpatient treatment program. After 4 weeks of abstinence, a 3-day experiment was conducted exposing participants to neutral, stress, or alcohol cue conditions using brief personalized imagery exposure, presented in randomized, counterbalanced order. Subjective craving, mood, cardiovascular function, salivary cortisol, and Stroop performance were assessed at baseline, immediately following imagery exposure, and at various recovery time points. Linear mixed-effects models tested ISEL scores as moderators.

**Results:**

Lower PSS was associated with higher nicotine craving during alcohol cue exposure and greater negative mood during stress. Both groups exhibited blunted cortisol responses to stress; however, individuals with lower perceived support showed greater attenuation in blood pressure responses. Higher perceived support was associated with better Stroop performance across conditions. No group differences were observed in recovery trajectories.

**Conclusions:**

PSS selectively moderates acute stress and cue reactivity during early abstinence, with lower support associated with a selective maladaptive behavioral and physiological profile. These findings suggest that social support confers modest, domain-specific resilience to acute psychological stress, highlighting its potential as a clinically relevant adjunct to more intensive treatment approaches during early abstinence from alcohol dependence.

## Plain Language Summary

In this study, we examined how perceived social support affects responses to stress and alcohol-related cues in people who are early in recovery from alcohol use disorder. Fifty-eight participants completed a laboratory experiment measuring mood, craving, and physical stress responses. Those with lower perceived support showed worse mood, higher nicotine craving, and more blunted blood pressure responses to stress and alcohol cues. However, social support did not normalize stress hormone responses. These findings suggest that social support provides selective benefits and may help improve emotional and cognitive functioning during early recovery.

In 2022, 29 million people in the United States were diagnosed with alcohol use disorder (AUD) (1), which is a leading preventable cause of death that continues to substantially impact health care resources (2,3) and workplace productivity (4, 5, 6) and perpetuate poverty (7, 8). Identifying resilience factors, which increase a person’s ability to adapt to stress (9), is key to developing effective individualized treatment targets. Because maladaptive biobehavioral stress-response systems during early abstinence from alcohol are associated with elevated craving and relapse (10, 11, 12), we sought to determine whether perceived social support (PSS) can act as a buffer against stress-system aberrations.

Social support relates to either perceived or actual emotional, instrumental, and/or informational support (13, 14, 15) that is available from various sources, including family, peers, partners, health care providers, and community members. Importantly, the stress-buffering effects of social support are purported to underpin resilience in high-stress drug- and alcohol-using populations (16, 17, 18, 19). Furthermore, the stress-buffering model of social support is founded on a plethora of research linking social support with a lower prevalence of disease (19,20), greater mental health benefits (21, 22, 23), prosocial decision-making (24), and overall longevity (25) via protection from stress-system pathophysiology (25,26). For example, in a recent review article, the authors concluded that heart rate variability, a biophysiological marker of the adaptive stress response, is associated with PSS during rest, stress induction, and recovery from an acute stressor (27). Similarly, low social support has been linked to elevated glucocorticoids and hypothalamic-pituitary-adrenal (HPA) axis hyperactivity (28), as well as elevations in inflammatory markers (29). High social support has also been shown to impact brain networks that downregulate stress systems (30), as well as buffer neural stress- and reward-circuit response to stress and cue exposure, respectively (31).

In this study, we used the Interpersonal Support Evaluation List (ISEL) (32) to assess PSS in a sample of inpatient treatment-seeking individuals with alcohol dependence during early abstinence (3–4 weeks). Given that self-perception is central to conceptualizations of loneliness, in which a discrepancy exists between one’s perceived and actual interpersonal relationships (32, 33, 34), we similarly adopted the construct of PSS to examine its role as a potential moderator of stress response. This approach is further supported by a substantial body of research demonstrating that PSS is a stronger predictor of improved mental health outcomes than other forms of social support (35).

Despite evidence linking PSS with resilience to stress-related pathophysiology, its impact on acute stress response during early abstinence from alcohol has not been systematically examined. This gap carries clinical significance because early abstinence represents a critical window during which acute withdrawal symptoms are resolved but subthreshold symptoms of incentive salience and stress response dysregulation persist (35,36). Furthermore, during this period, sensitized negative mood and craving, alongside hyporesponsivity of the HPA axis and autonomic nervous system, are widely considered central mechanisms linking stress responses to alcohol-seeking behavior (37, 38, 39). Therefore, identifying clinical moderators of the stress response during this period may inform the development of more targeted interventions, particularly by establishing the extent to which social support-focused approaches could help ameliorate stress and craving in an ecologically valid inpatient setting.

We hypothesized that during early abstinence from alcohol dependence, individuals reporting lower PSS would exhibit greater emotional and biophysiological stress-response dysregulation, characterized by heightened stress-induced alcohol craving and negative mood and/or greater attenuation of cortisol and cardiovascular reactivity.

## Methods and Materials

### Participants

We analyzed data collected from *N* = 58 treatment-seeking individuals who were originally diagnosed with alcohol dependence using the Structured Clinical Interview for DSM-IV (SCID-IV) (40). Participants were recruited through advertisements and flyers around the local New Haven, Connecticut, area into 4 weeks of inpatient stay and study participation at the Clinical Neuroscience Research Unit (CNRU) of the Connecticut Mental Health Center (CMHC). Exclusion criteria included DSM-IV-TR dependence on any substance other than alcohol or nicotine and any psychiatric or chronic medical condition requiring medication, except for stable use of selective serotonin reuptake inhibitors. Prior to enrollment, all participants underwent a standardized battery of medical screening tests, comprising a complete blood count, erythrocyte sedimentation rate, fasting glucose, blood urea nitrogen/creatinine, serum electrolytes, liver function tests, thyroid function tests, and an electrocardiogram. Participants whose results fell outside the standard reference ranges were referred for further clinical assessment by the study physician, who determined on a case-by-case basis whether the individual remained eligible to participate in the research component of the study. Following admission procedures, baseline measures were administered to determine any potential statistical covariates, including demographics, alcohol and substance use (41), and psychiatric status as assessed by the SCID-IV (40). In addition, the ISEL was administered to determine PSS.

During week 4 of their inpatient stay, all participants took part in 3 laboratory challenge studies, conducted across 3 consecutive days, during which they were presented with 3 personalized 5-minute imagery conditions (stress, alcohol cue, and neutral), one per day, in a randomized and counterbalanced order. Staff and participants were both blinded to the presentation order. Personalized imagery scripts were developed and scripted during week 2 of the inpatient stay from participants’ recent life events (42).

### Determining PSS

The ISEL is a 40-item scale that can be used to determine the perceived availability of 4 separate functions of social support in addition to a total PSS metric. All items are equally divided into either positive or negative statements regarding social relationships, and participants indicate whether these statements are “probably true” or “probably false” for them. The highest possible total score is 40, 10 for each of the 4 subscales, and the higher the score, the greater the perceived social support. The subscales comprise: 1) a “tangible support” scale assessing the perceived availability of material aid (e.g., someone to help with practical tasks), 2) an “appraisal support” scale reflecting the availability of someone with whom to discuss problems (e.g., someone to talk to about personal concerns), 3) a “self-esteem support” scale capturing positive social comparison (e.g., feeling valued relative to others), and 4) a “belonging support” scale indicating the perceived availability of social companionship (e.g., someone to spend time with). The ISEL demonstrates strong psychometric properties (42, 43, 44) and has been validated in diverse populations and cultural groups (45, 46, 47).

### Script Development

Imagery script development procedures are fully described in Sinha and Tuit (42). Briefly, participants provided 3 personalized scenarios that had occurred during the past year. These included a neutral or relaxing scenario, a stress scenario, and an alcohol cue-related scenario. Stress scripts were based on descriptions of recent personal stressful events that were experienced as “most stressful” on a 10-point Likert scale, where 1 = not at all stressful and 10 = the most stress they had felt recently in their life, and only situations rated as ≥8 were accepted as appropriate for script development. The stress scenarios could not be related to drinking or result in drinking. Conversely, the alcohol cue scripts were developed by having participants identify recent situations that included alcohol-related stimuli and resulted in subsequent alcohol use (e.g., being at a bar, watching others drink alcohol) but were not stressful. While no stressful information was included in the alcohol cue scenarios, our previous research has shown that the alcohol cue script reliably elicits a state of elevated negative affect in this inpatient population (48, 49, 50) and therefore is considered a variant stressor. The neutral scripts comprised a relaxing script developed from descriptions of a personal event, such as being at the beach or spending a fall afternoon reading at the park, without the inclusion of alcohol-related stimuli. All scripts also included descriptions of bodily sensations (e.g., muscle tension, perspiration), which participants chose from a checklist of interoceptive sensations that they experienced in the described scenarios. All guided imagery scripts were written by a clinician and recorded onto an audiotape to be played to participants across 3 consecutive days, 1 image per day, in a randomized and counterbalanced order.

### Laboratory Sessions

During the week prior to the laboratory sessions, participants received imagery and relaxation training to increase consistency during the 3 laboratory sessions. On each testing day, subjects were brought into the testing room at 8:00 am. All subjects were allowed an initial smoke break immediately prior to this to reduce nicotine craving. After settling into a sitting position on a hospital bed, all participants were allowed a 30-minute adaptation period during which they were instructed to practice relaxation. At 8:30 am, participants were provided with headphones and given the following instructions: “Close your eyes and imagine the situation being described ‘as if’ it were happening right now. Let your body and mind get completely involved in the situation, doing what you would do in the real situation.” The length of each imagery script was 5 minutes. Subjective measures of alcohol craving, nicotine craving, and mood were collected, in addition to heart rate and blood pressure, as well as saliva samples of cortisol, 5 minutes prior to imagery exposure (baseline), immediately following imagery exposure, and periodically at various recovery time points (+5, +15, +30, +45, +60 + 75 minutes post-imagery). The Stroop Color-Word Test was administered at baseline and +5 minutes following imagery exposure only.

### Laboratory Assessments

Subjective measures included alcohol/nicotine craving, which was defined as the desire for use either alcohol or nicotine as expressed using a 10-point visual analog scale in which 1 = “not at all” and 10 = “extremely high.” Mood was calculated using the Differential Emotion Scale (51). Participants were required to rate, on a 5-point scale, the extent to which a series of adjectives described the way they felt at that time. Subscales included anger, fear, sadness, anxiety, joy, and relaxed feelings. Negative mood was calculated using a composite score of anger, sadness, fear, and anxiety, and positive mood was calculated using a composite score of joy and relaxed feelings.

### Cardiovascular Measures

An SD-700A Monitor (IBS Corp.) was used to assess blood pressure. A pulse sensor was attached to the subject’s finger and connected to the SD-700A Monitor to provide a continuous measure of pulse.

### Salivary Measures

Saliva samples were collected using standard procedures, stored at −20 °C, and assayed in duplicate using commercially available radioimmunoassay kits at the Yale General Clinical Research Center’s Core Laboratories. The intra-assay coefficients of variation range from 3.0% to 5.1%.

### Stroop Performance

The Stroop Color-Word Test (52) was used to assess selective attention and cognitive control. Participants completed three 45-second trials involving word reading, color naming, and color-word interference, with scores reflecting correct responses while suppressing the tendency to read the word.

### Data Analyses

Linear mixed-effects (LME) models were used to analyze baseline and change from baseline data using SPSS Statistics, version 31 (IBM Corp.) (53). The between-subjects factor of PSS (high PSS [HPSS] and low PSS [LPSS]) based on a median split of ISEL scores and the within-subjects factors of imagery condition (stress, neutral, alcohol cue) and time points (varying levels) represented the fixed-effects factors. Participants represented the random-effects factor. Bonferroni correction was applied to comparisons within each outcome. The HPSS and LPSS groups were compared on demographics and alcohol use using *t* tests or χ2 tests (Table 1). Because cannabis use differed between the groups (Table 1), the number of days of use in the past month was included as a covariate in the mixed-effects models.

**Table 1:** Demographics and Alcohol/Substance Use by High and Low Perceived Social Support Group

Demographics	High Perceived Social Support, *n* = 33	Low Perceived Social Support, *n* = 25	*p* Value
Age, Years	37.3 ± 8.4	37.8 ± 8.3	n.s.
Sex			
Female	8 (24%)	5 (20%)	n.s.
Male	25 (76%)	20 (80%)	
Race			
African American	8 (24%)	6 (24%)	n.s.
Asian	0 (0%)	1 (4%)	
Caucasian	23 (70%)	18 (72%)	
Hispanic	2 (6%)	0 (0%)	
Alcohol			
Years of alcohol use	18.7 ± 8.3	17.4 ± 8.9	n.s.
Alcohol use in the 30 days prior to treatment, no. of drinks	500.4 ± 555	371.0 ± 289	n.s.
Alcohol use in the 30 days prior to treatment, no. of days	18.6 ± 10.2	19.0 ± 9.4	n.s.
No. of times treated for alcohol use	5.5 ± 10.1	7.8 ± 11.1	n.s.
No. of times treated for detox only	3.1 ± 10.8	2.5 ± 4.9	n.s.
Other Substances			
No. of smokers	28 (85%)	24 (96%)	n.s.
Cocaine use in the 30 days prior to treatment, no. of days	5.6 ± 9.5	4.0 ± 6.9	n.s.
Cannabis use in the 30 days prior to treatment, no. of days	3.3 ± 8.7	0.7 ± 2.1	*p* = .05
Psychiatric			
Lifetime mood disorder	4 (12%)	4 (16%)	n.s.
Current mood disorder	1 (3%)	1 (4%)	n.s.
Lifetime PTSD	5 (15%)	2 (8%)	n.s.
Current PTSD	4 (12%)	1 (4%)	n.s.
Lifetime anxiety w/o PTSD	0 (0%)	4 (16%)	n.s.
Current anxiety w/o PTSD	0 (0%)	1 (4%)	n.s.

Values are mean ± SD or *n* (%).

n.s., not significant; PTSD, posttraumatic stress disorder; w/o, without.

Median-split analyses are presented for visualization and interpretability purposes (HPSS: ≥33; LPSS: <33 on the ISEL); however, interactions were interpreted only when a corresponding significant interaction was observed using PSS as a continuous moderator in the LME models. Slope graphs from the continuous ISEL analyses, in which scores were mean-centered, are provided in the Supplement and are consistent with the median split findings.

Main effects of PSS group (high and low), imagery condition (stress, alcohol cue, and neutral), and their interactions are presented in the text. All simple-effects analyses for imagery × time point interactions are reported in the Supplement (Table 1).

## Results

### Baseline

A main effect of PSS was shown for salivary cortisol (*F*_1,54_ = 4.2, *p* < .05), wherein the LPSS group demonstrated significantly lower levels of cortisol than the HPSS group.

### Effect of Imagery: Craving and Mood (Main Effects)

A main effect of imagery condition was observed for alcohol craving (*F*_2,1077_ = 55.5, *p* < .001), nicotine craving (*F*_2,1077_ = 68.2, *p* < .001), and negative mood (*F*_2,1077_ = 92.0, *p* < .001). Higher ratings were reported in all participants in the stress and alcohol cue conditions than in the neutral condition (*p* < .001, in all cases). Higher alcohol craving was also reported in the alcohol cue than in the stress condition (*p* = .002), and higher nicotine craving and negative mood were reported in the stress than in the alcohol cue condition (*p* < .001, in all cases). A main effect of imagery condition for positive mood (*F*_2,1077_ = 90.6, *p* < .001) also indicated that all participants reported higher positive mood in the neutral than in the stress and the alcohol cue conditions, as well as in the alcohol cue condition compared with the stress condition (*p* < .001, in all cases). All measures showed a significant main effect of time point (*F*_6,1077_ = 5.9, all *p*s < .001), reflecting elevated negative mood and craving immediately following imagery exposure with subsequent recovery and a corresponding decrease in positive mood that improved over time.

### Subjective Measures of Craving and Mood (Interaction Effects)

**Figure 1:**
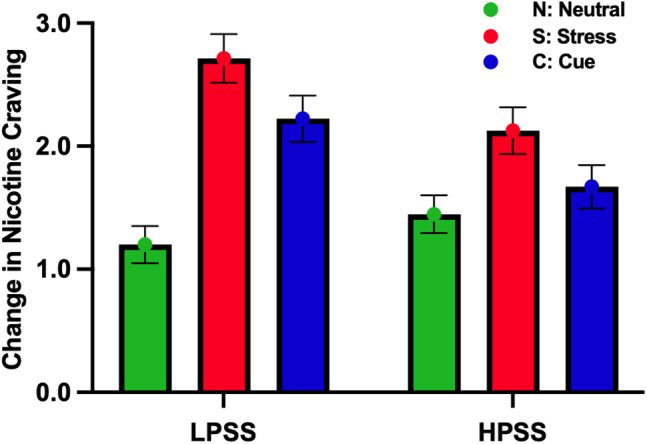
Nicotine craving in high and low perceived social support (PSS) groups across all 3 imagery conditions. Within-group differences: in low PSS (LPSS): stress > neutral, *p* < .001; stress > alcohol cue, *p* = .002; alcohol cue > neutral, *p* < .001. In high PSS (HPSS): stress > neutral, *p* < .001; stress > alcohol cue, *p* < .001. Bars represent mean ± SEM.

**Figure 2:**
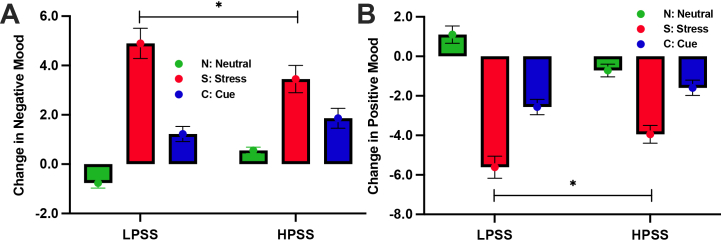
**(A)** Negative mood in high and low perceived social support (PSS) groups across all 3 imagery conditions. Between-group differences: displayed on graphs (∗*p* < .05). Within-group differences: in low PSS (LPSS): stress > neutral, *p* < .001; stress > alcohol cue, *p* < .001; alcohol cue > neutral, *p* < .001. In high PSS (HPSS): stress > neutral, *p* < .001; stress > alcohol cue, *p* < .001; alcohol cue > neutral, *p* = .007. Bars represent mean ± SEM. **(B)** Positive mood in high and low perceived social support groups across all 3 imagery conditions. Between-group differences: displayed on graphs (∗*p* < .05). Within-group differences: LPSS: neutral > stress, *p* < .001; neutral > alcohol cue, *p* < .001; alcohol cue > stress, *p* < .001. In HPSS: neutral > stress, *p* < .001; alcohol cue > stress, *p* < .001. Bars represent mean ± SEM.

Significant PSS group × imagery condition interactions were observed for alcohol craving (*F*_2,1077_ = 3.1, *p* = .04), nicotine craving (*F*_2,1077_ = 11.7, *p* < .001), negative mood (*F*_2,1077_ = 10.3, *p* < .001), and positive mood (*F*_2,1077_ = 12.4, *p* < .001). Post hoc analyses indicated that while both PSS groups reported higher nicotine craving following stress (*p* < .001, in both cases), only the LPSS group reported higher nicotine craving in the alcohol cue than in the neutral condition (*p* < .001). This was not observed in the HPSS group (Figure 1). The LPSS group also reported significantly higher negative mood (*p* = .05) and lower positive mood following exposure to stress (*p* < .05) than the HPSS group (Figure 2A, B). Conversely, participants in the LPSS group also reported greater positive mood on average during exposure to the neutral condition than participants in the HPSS group (*p* = .02). While the group × imagery condition interaction for alcohol craving was significant in the median split analysis, it was not supported by the continuous moderation analysis and therefore is not interpreted further.

No significant interactions between PSS group × time point were found for any of the subjective measures. However, significant imagery × time point interactions emerged across all measures, suggesting that participants’ craving and mood responses followed a similar pattern regardless of their level of perceived social support, with comparable reactivity to stress and alcohol cue imagery compared with neutral imagery and a similar recovery trajectory.

### Cardiovascular Response (Interaction Effects)

**Figure 3:**
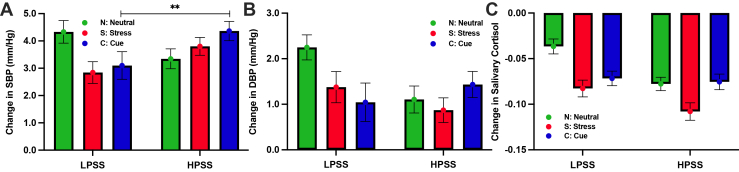
**(A)** Systolic blood pressure (SBP) in high and low perceived social support (PSS) groups across all 3 imagery conditions. Between-group differences: displayed on graphs (∗∗*p* < .01). Within-group differences: in low PSS (LPSS): neutral > alcohol cue, *p* = .05. In high PSS (HPSS): alcohol cue > neutral, *p* = .05. Bars represent mean ± SEM. **(B)** Diastolic blood pressure (DBP) in high and low PSS groups across all 3 imagery conditions. Within-group differences: In LPSS: neutral > alcohol cue, *p* = .009. Bars represent mean ± SEM. **(C)** Salivary cortisol in high and low PSS groups across all 3 imagery conditions. Within-group differences: in both groups, neutral > stress, *p* < .001; alcohol cue > stress, *p* = .004. Bars represent mean ± SEM. The blunted response in the stress condition is demonstrated by the continued diurnal drop in cortisol across the course of the morning.

A significant PSS group × imagery condition interaction was observed for systolic blood pressure (SBP) (*F*_2,1233_ = 5.6, *p* = .004). Post hoc analysis indicated that the HPSS group demonstrated higher SBP in the alcohol cue condition than the LPSS group (*p* < .01). The LPSS group also showed lower SBP in the alcohol cue than in the neutral condition (*p* = .05). Conversely, in the HPSS group, response to alcohol cues was greater than response to neutral cues (*p* = .05; Figure 3A). A significant PSS group × imagery condition interaction was also seen for diastolic blood pressure (DBP) (*F*_2,1233.4_ = 4.5, *p* = .01) showing that the LPSS group demonstrated lower DBP in the alcohol cue condition than in the neutral imagery condition (*p* = .009). This dampened response was not observed in the HPSS group (Figure 3B). No main effects of PSS group or imagery condition were observed. Also, no time point × imagery or time point × group interactions were seen. A main effect of time point was seen for DBP indicating that the DBP response immediately following all 3 imageries was greater than at later time points. Complete pairwise comparisons are reported in Table S1. No significant group main effects or group × imagery interactions were observed for heart rate.

### Salivary Cortisol

A main effect of imagery condition (*F*_2,770.6_ = 9.8, *p* < .001) indicated that all participants, irrespective of PSS, demonstrated significantly lower cortisol levels following stress imagery exposure compared to both neutral (*p* < .001) and alcohol cue imagery exposure (*p* = .004; Figure 3C). The main effect of time point (*F*_5,760.9_ = 13.1, *p* < .001) also reflected elevated cortisol following imagery exposure with subsequent decreases across time.

### Stroop Color-Word Performance

**Figure 4:**
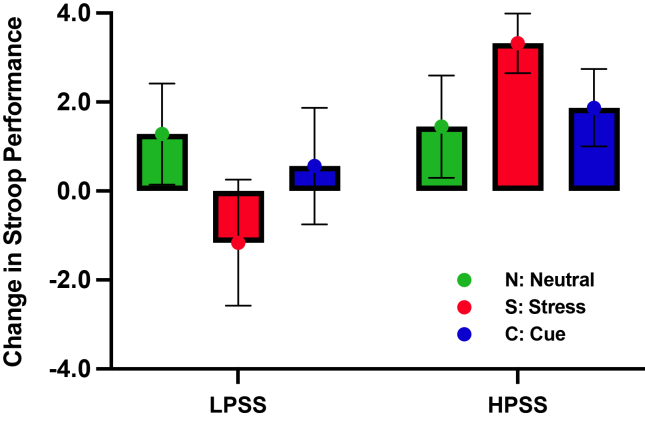
Stroop Color-Word performance in high and low perceived social support (PSS) groups across all 3 imagery conditions. Main effect of group: high PSS (HPSS) > low PSS (LPSS), *p* < .05. Bars represent mean ± SEM.

A main effect of PSS group (*F*_1,53_ = 4.1, *p* < .05) indicated that the HPSS group performed significantly better on the Stroop than the LPSS group across all 3 imagery conditions (Figure 4).

## Discussion

Collectively, these findings indicate that PSS exerts a domain-specific rather than a global stress-buffering effect during early abstinence from alcohol. Social support represents a key resilience factor in the development and outcome of alcohol and other substance use disorders (54, 55, 56, 57). Because this may be due in part to social support acting as a buffer against stress-system pathophysiology (26,55), we investigated the moderating effects of PSS in response to acute psychological stress in treatment-seeking individuals with alcohol dependence during early abstinence.

While PSS had no impact on stress- or cue-induced alcohol craving, individuals with higher PSS reported lower nicotine craving in response to alcohol cues. This suggests that while PSS may confer some protection against cross-cue, or secondary, nicotine craving reactivity to alcohol cues, it may not be adequate to curtail primary alcohol craving during the early abstinence period of inpatient treatment. Given that all participants, irrespective of their level of PSS, were undergoing inpatient treatment for alcohol dependence, it is possible that perceived psychosocial factors alone may be insufficient to meaningfully reduce primary alcohol craving during early abstinence. However, findings should also be interpreted in the context of nicotine use, which may act as a concurrent modulator of cue reactivity and represents an important target for future investigation.

PSS modified affective responses to stress, with higher support associated with lower stress-induced negative mood and higher positive mood. Although elevations in negative mood are often associated with relapse (57, 58, 59), evidence also suggests that this relationship is mediated by increases in craving (60). In contrast, however, the current findings demonstrate a dissociation between these domains such that perceived social support buffered affective responses to stress but did not alter alcohol craving. Similarly, while PSS attenuated cue-related nicotine craving, no corresponding mood effects were observed. This dissociation suggests that stress-induced craving may, in certain circumstances, be driven by mechanisms that are partially independent of affective state and elicited directly by the stressor itself via habituated pathways that are dissociable from the affective regulatory system (60,61).

With respect to biophysiological responses, effects were again selective, and benefits were confined to the alcohol cue condition. Individuals with HPSS demonstrated a relatively preserved blood pressure response, whereas those with lower support exhibited a blunted response. This pattern is consistent with the reduced cardiac responsiveness to stress that has been observed in certain alcohol-using populations (62,63). Notably, however, the response of other biophysiological markers remained unchanged by PSS, where both groups demonstrated dysregulated blunted cortisol responses to stress, and neither group showed significant heart rate elevations following stress or alcohol cues, indicating broader hyporesponsivity of stress systems that were not modified by PSS.

Importantly, the convergence of blunted cortisol and blood pressure responses may reflect reduced engagement of both HPA axis and autonomic stress systems and is consistent with a broader stress-system attenuation phenotype. This pattern of autonomic and HPA axis hyporesponsivity is well-documented across diverse stressors in individuals with AUD (64) and other substance use disorders, including nicotine, cannabis, and opioid dependence (65, 66, 67) as well as a marker of allostatic dysregulation and a maladaptive phenotype associated with relapse (9,65, 66, 67, 68, 69, 70). Notably, blunted cardiovascular output has also been linked to behaviors that overlap with perceived social support, including motivational disengagement and depression (71).

Unlike the mood and cardiovascular findings, the Stroop performance advantage observed in individuals with HPSS was present across all conditions, including neutral. This suggests that the cognitive benefits of PSS may reflect a general regulatory advantage rather than a stress-specific buffering effect, consistent with the direct effect model of social support (72).

Several methodological limitations warrant consideration. First, the small sample size and low proportion of female participants limit generalizability beyond alcohol-dependent men and constrain the interpretation of moderation effects. Second, while social support was dichotomized using a median split to reflect how this construct is typically conceptualized clinically (as a perceived presence or absence of support) this approach carries inherent limitations, including potential loss of individual difference information. To address this, all moderation analyses were first conducted using perceived social support as a continuous variable, with median split interactions interpreted only where a corresponding significant interaction was observed in the continuous model, thereby ensuring reported effects were supported by the more statistically powerful analysis. Third, all laboratory sessions were conducted at 8:00 am to minimize daily confounds within the inpatient environment, including food intake, counseling sessions, and unpredictable psychosocial stressors. However, this timing may have attenuated provoked craving responses given that alcohol-dependent individuals are typically less likely to drink in the early morning, and cortisol reactivity may differ at other points in the diurnal cycle when the cortisol waking response is less pronounced. Future studies would benefit from replicating these assessments at alternative times during the day to determine whether the observed patterns generalize across the diurnal rhythm.

These findings suggest that perceived social support exerts selective, domain-specific effects on stress and cue reactivity during early alcohol abstinence, rather than functioning as a global stress buffer. However, PSS did not appear to normalize HPA axis and autonomic hyporesponsivity, and biobehavioral responses across recovery time points remained similar in both HPSS and LPSS groups. Together, these findings suggest that social support-focused interventions may offer meaningful clinical benefit for treatment-seeking individuals in early alcohol abstinence, particularly when delivered as an adjunct to more intensive treatment approaches.

## Supplementary Material

Figures S1–S5 and Table S1

Key Resources Table
