# Supplementary material for: Perceived Social Support Moderates the Response to Stress and Alcohol Cues During Early Abstinence From Alcohol
Source: Biol Psychiatry Glob Open Sci. 2026 May 28;6(5):100762. doi: 10.1016/j.bpsgos.2026.100762 (PMC13382264; doi:10.1016/j.bpsgos.2026.100762)
Supplement: Figures S1–S5 and Table S1 [file mmc1.pdf]

SUPPLEMENTARY INFORMATION

Perceived Social Support Moderates the Response to Stress and Alcohol Cues During Early Abstinence from Alcohol

Goldstein *et al.*

Supplementary Material

Table S1. Main Effects and Timepoint (TP) × Imagery Interactions for Craving, Mood, and Physiological Outcomes

Measure	Main Effects & Interactions	Simple Effects
**Alcohol Craving**	**Timepoint:** [F(6, 1077) = 48.4, P < .001]<br>**Timepoint × Imagery:** [F(12, 1077) = 12.7, P < .001]	**Overall Time Effects:**<br>• 0TP > all (P < .001)<br>• +5TP > +15, +30, +45, +60, +75 (P = .04)<br>**Neutral:** no significant differences<br>**Stress:** 0TP > all (P < .001); +5TP > +45TP, +60TP, +75TP<br>**Alcohol Cue:** 0TP > all (P < .001); +5TP > +15TP, +30TP, +45TP, +60TP
**Nicotine Craving**	**Timepoint:** [F(6, 1077) = 5.9, P < .001]<br>**Timepoint × Imagery:** [F(12, 1077) = 1.9, P = .03]	**Overall:**<br>• 0TP > +5TP (trend)<br>• +65TP, +75TP > +15TP (P = .009)<br>**Neutral:** 0TP > +60, +75<br>**Stress:** 0TP > +15<br>**Alcohol Cue:** 0TP > +5 (trend)
**Negative Mood**	**Timepoint:** [F(6, 1077) = 92.2, P < .001]<br>**Timepoint × Imagery:** [F(12, 1077) = 22.3, P < .001]	**Overall:**<br>• 0TP > all (P < .001)<br>• +5TP > +30, +45, +60, +75<br>**Neutral:** no significant differences<br>**Stress:** 0TP > all (P < .001)<br>**Alcohol Cue:** 0TP > all (P < .001)
**Positive Mood**	**Timepoint:** [F(6, 1077) = 8.3, P < .001]<br>**Timepoint × Imagery:** [F(12, 1077) = 12.6, P < .001]	**Overall:**<br>• 0TP > +30, +45, +60, +75<br>**Neutral:** 0TP > all<br>**Stress:** 0TP < all (P < .001)<br>**Alcohol Cue:** no significant differences
**Heart Rate**	**Timepoint:** [F(7, 1233) = 11.0, P < .001]<br>**Timepoint × Imagery:** [F(14, 1233) = 2.6, P = .001]	**Overall:**<br>• 0TP > all<br>• +5TP, +15TP > +30TP, +60TP<br>**Neutral:** +5, +15 > +30<br>**Stress:** 0TP > all (P < .001)<br>**Alcohol Cue:** 0TP > +60
**Diastolic Blood Pressure**	**Timepoint:** [F(7, 1233) = 2.8, P = .007]<br>**Timepoint × Imagery:** [F(14, 1233) = 1.1, P = .37]	**Overall:**<br>• 0TP > +15TP, +75TP<br>**Neutral:** no significant differences<br>**Stress:** no significant differences<br>**Alcohol Cue:** no significant differences
**Systolic Blood Pressure**	**Timepoint:** [F(7, 1233) = 0.54, P = .81]<br>**Timepoint × Imagery:** [F(14, 1233 = .59, P = .87]	**Overall:**<br>• no significant effects<br>**Neutral:** no significant differences<br>**Stress:** no significant differences<br>**Alcohol Cue:** no significant differences
**Salivary Cortisol**	**Timepoint: [**F(5, 761) = 13.1, *p* < .001]<br>**Timepoint × Imagery:** [F(10, 761) = 0.52, P = .88]	**Overall:**<br>• 0TP > +15TP, +30TP, +45TP, +60TP, +75TP<br>• +15TP > +60TP<br>**Neutral:** no significant differences<br>**Stress:** no significant differences<br>**Alcohol Cue:** no significant differences

TP, Timepoint.

Figure S1. Change in Systolic Blood Pressure (SBP) by Total Perceived Support as Assessed by the Interpersonal Support Evaluation List (ISEL)

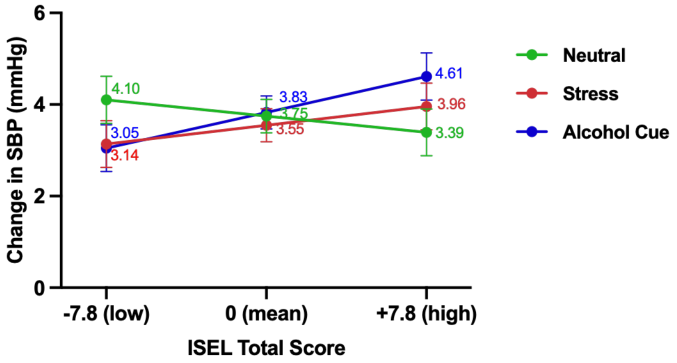

The graph displays the interaction between perceived social support as assessed by ISEL and change in SBP from baseline, plotted at three levels of the mean-centered social support moderator: −1 SD (low), mean, and +1 SD (high). The graph is consistent with the median split analyses showing lower SBP in the stress relative to neutral imagery condition in individuals with low perceived social support (LPSS; (P = .03). It also demonstrates the significantly elevated SBP in stress (P = .05) and alcohol cue (P = .05) in the high perceived social support group (HPSS) group compared with the LPSS group.

Figure S2. Change in Diastolic Blood Pressure (DBP) by Total Perceived Support as Assessed by the ISEL

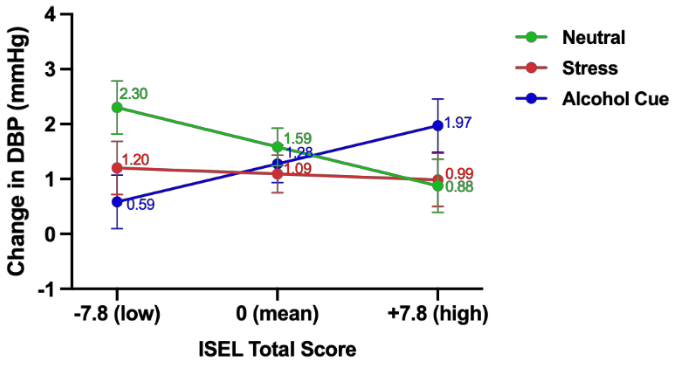

The graph displays the interaction between perceived social support and change in diastolic blood pressure from baseline, plotted at three levels of the mean-centered social support moderator: −1 SD (low), Mean, and +1 SD (high). This is consistent with the median split analyses, where individuals with LPSS (−1 SD) showed significant lower DBP in the alcohol cue condition relative to neutral (P < .02), whereas no such dampening was observed in individuals with HPSS (+1 SD).

Figure S3. Change in Nicotine Craving by Total Perceived Support as Assessed by the ISEL

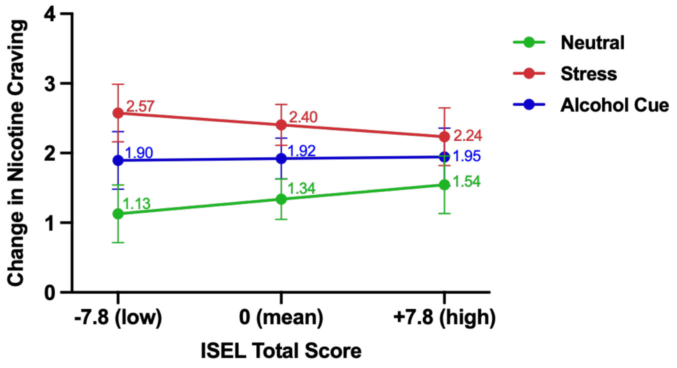

The graph displays the interaction between perceived social support and change in nicotine craving from baseline, plotted at three levels of the mean-centered social support moderator: −1 SD (low), mean, and +1 SD (high). This is consistent with the median split analyses, which showed no significant elevation in nicotine craving in the alcohol cue relative to the neutral condition in individuals with HPSS, whereas individuals with LPSS showed a significant cue-induced elevation ($P < .001$)

Figure S4. Change in Negative Mood by Total Perceived Support as Assessed by the ISEL

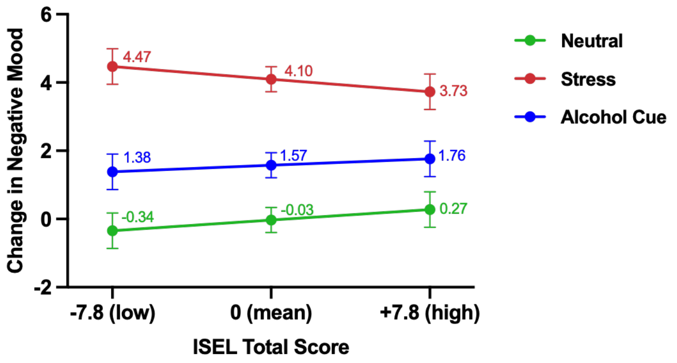

The graph displays the interaction between perceived social support and change in negative mood from baseline, plotted at three levels of the mean-centered social support moderator: −1 SD (low), mean, and +1 SD (high). This is consistent with the median split analyses, where individuals with LPSS (-1 SD) showed a significant elevation in negative mood in the stress relative to neutral condition ($P = .02$), which was not observed in the individuals with HPSS (+1 SD).

Figure S5. Change in Positive Mood by Total Perceived Support as Assessed by the ISEL

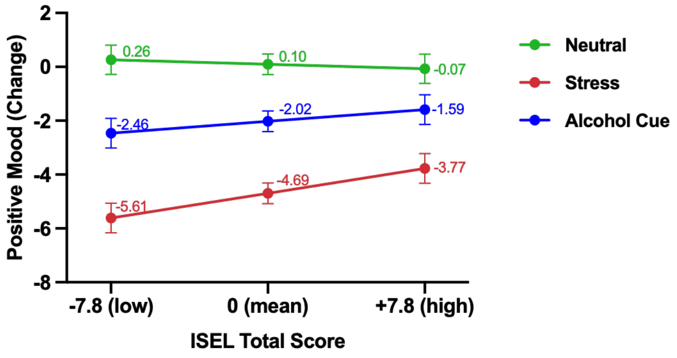

The graph displays the interaction between perceived social support and change in positive mood from baseline, plotted at three levels of the mean-centered social support moderator: −1 SD (low), mean, and +1 SD (high). This is consistent with the median split analyses, where individuals with LPSS (-1 SD) reported lower positive mood following stress imagery relative to individuals with HPSS (+1 SD; $P < .05$).
